# Environmental severe acute respiratory syndrome coronavirus 2 (SARS-CoV-2) contamination in hospital rooms during the first and third coronavirus disease 2019 (COVID-19) waves

**DOI:** 10.1017/ash.2022.228

**Published:** 2022-05-20

**Authors:** Killian Le Neindre, Jeanne Couturier, Aurélie Schnuriger, Sarah Jolivet, Cyril Gouot, Mickaël Majerholc, Pierre Supplisson, Céline Tan, Marine Perrier, Christelle Lazare, Laurence Morand-Joubert, Frédéric Barbut

**Affiliations:** 1 Department of Environmental Microbiology, Saint-Antoine Hospital, Sorbonne Université, Assistance Publique – Hôpitaux de Paris (AP-HP), Paris, France; 2 Infection Control Unit, Saint-Antoine Hospital, Sorbonne Université, Assistance Publique – Hôpitaux de Paris (AP-HP), Paris, France; 3 Department of Virology, Saint-Antoine Tenon Trousseau Hospitals, Sorbonne Université, Assistance Publique – Hôpitaux de Paris (AP-HP), France; 4 Department of Virology, Sorbonne Université, INSERM, Institut Pierre Louis d’Epidémiologie et de Santé Publique (iPLESP), Assistance Publique-Hôpitaux de Paris (AP-HP), Paris, France

## Abstract

We investigated the frequency, distribution, and risk factors of severe acute respiratory syndrome coronavirus 2 (SARS-CoV-2) environmental contamination around infected patients during the first and third wave of the coronavirus disease 2019 pandemic. The shedding of SARS-CoV-2 in rooms of infected patients was limited in our hospital setting.

Since December 2019, the severe acute respiratory syndrome coronavirus 2 (SARS-CoV-2) has disseminated worldwide, causing an unprecedented public health threat. SARS-CoV-2 is primarily transmitted directly from person to person by respiratory transmission.^1^ In the hospital setting, SARS-CoV-2 is rarely detected in air samples, and when it is detected the viral load is very low.^2^ Surfaces could also be involved in SARS-CoV-2 transmission. Surface contamination is highly variable across studies and wards,^3^ and SARS-CoV-2 has been shown to persist on different surfaces from several hours to days.^4,5^ The factors associated with environmental contamination are still unclear and require further investigation. In this study, the frequency of surfaces and air contamination by SARS-CoV-2 was evaluated in rooms of patients infected by SARS-CoV-2 and risk factors associated with the environmental contamination were investigated.

## Methods

### Study design

This prospective study was conducted in a university-affiliated, 650-bed, acute-care hospital in France during the end of the first epidemic wave (April to August 2020) and during the third epidemic wave (February–May 2021). Symptomatic patients were screened for coronavirus disease 2019 (COVID-19) on admission except for 19 patients who came to the hospital with an external positive test. Rooms of patients infected by SARS-CoV-2 were sampled at least 4 hours after the cleaning procedure and within 15 days following the first positive biological diagnosis by reverse-transcription quantitative polymerase chain reaction (RT-qPCR). The cleaning procedure was manual using a detergent disinfectant product (containing quaternary ammonium compound). Environmental sampling included 4–10 surfaces (mainly door handle, respirator, syringe driver, bed rail, floor, bench, fluids ramp, bedside table, toilet seat, sink, and windowsill) and air.

For each patient, the following data were collected: age, sex, symptoms at the time of sampling, type of ward (medicine or intensive care unit), type of mechanical ventilation, delay between positive diagnosis and environmental sampling, origin of SARS-CoV-2 infection (community versus hospital acquired), type of SARS-CoV-2 variant (when available) and cycle threshold (Ct) values. We defined a hospital-acquired infection as a patient admitted without symptoms suggestive of COVID-19 who had SARS-CoV-2 RNA detected at least 3 days after admission.

### Environmental sampling and virology assays

Surfaces (10-cm × 10-cm) were sampled using premoistened swabs discharged in 500 µL viral medium transport VTM (Labomoderne, France). Air (1,000 L, 50 L/min flow) was sampled 1 m from the patient using the MD8 air sampler (Sartorius, Germany). Air was collected onto gelatin membrane filters (Sartorius, Germany) that were dissolved in 10 mL sterile water at 37°C for 10 minutes. Environmental samples were analyzed by reverse-transcription quantitative polymerase chain reaction (RT-qPCR) using the Allplex SARS-CoV-2 Assay (Seegene, South Korea) or Bosphore Novel Coronavirus (2019-nCoV) Detection Kit v2 (Anatolia Genework, Turkey). The Allplex kit (SEE) amplified the *E* gene, *RdRP/S* gene, and *S* gene, whereas the Bosphore kit (BOS) amplified the *E* gene and *orf1ab* gene. Positive results were presented as the mean of PCR cycle threshold (Ct) values for each viral genes. Both techniques yield similar Ct values, as previously described.^6^

### Statistical analysis

A room was considered contaminated when at least 1 environmental sample was positive. Contaminated and noncontaminated rooms were compared using the Fisher exact test or the χ^2^ for qualitative data and the Mann-Whitney test for quantitative data. Statistical tests were performed using GraphPad Prism version 7 software (GraphPad Software, San Diego, CA).

## Results

In total, 61 rooms were sampled, and 338 surfaces and 59 air samples were analyzed (Table 1). The median age of patients was 73 years (interquartile range [IQR], 61–84), and the sex ratio (M/F) was 2.2. The median delay between symptom onset and environmental sampling was 9 days (IQR, 6–13). Environmental sampling was conducted after a median delay of 4 days (IQR, 2–7) following admission and after a median delay of 3 days (IQR, 2–7) following positive RT-qPCR. Identification of variants was not performed during the first wave.

**Table 1.** Description of Data Collected From Rooms With Positive Environmental Samples

Room	Date of Sampling	Ward	Ct Value (Clinical)	SARS-CoV-2 Variant	Mechanical Ventilation	Oxygen Flow, L/min	Positive Surface Sample/Total Surface	Ct per Sample (Environmental)
1	04-20	ICU	Positive	NA	Intubation	NA	3/10	36.03/37.43/37.74
2	04-20	MD	26.04	NA	Nasal	2	3/10	39.03/35.52/37.84
3	06-20	MD	26.95	NA	Absence	No	4/10	38.72/36.97/37.1/38.6
7	07-20	MD	12.47	NA	Mask	13	2/10	36.77/38.16
15	02-21	MD	20.18	H	Nasal	2	2/4	38.33/38.08
16	02-21	MD	28.83	H	Absence	No	0/4	37.66^a^
25	02-21	MD	19.97	α	Absence	No	1/4	30.35
29	03-21	MD	26.46	H	Absence	No	1/4	28.60
49	04-21	MD	23.62	α	Absence	No	1/5	40
52	04-21	MD	29.78	α	Absence	No	3/5	34.13/30.34/33.74
53	04-21	MD	33.02	β	Mask	7	1/5	39.18
54	04-21	MD	11.81	β	Nasal	5	1/5	35.66
55	04-21	MD	Positive	NA	Mask	12	1/5	35.74
61	05-21	ICU	20.78	β	Optiflow	60	1/5	38.07

Note. Ct, cycle threshold; MD, medicine; ICU, intensive care unit; M, male; F, female; Positive: positive test but the Ct value was not available; H, historical strain;

NA, data not available.

a Ct from positive air sample.

Of 61 rooms, 14 (22.9%) had at least 1 positive sample for SARS-CoV-2 (Table 1). Overall, 24 (7.1%) of 338 surfaces and 1 (1.7%) of 59 air samples were positive. Cycle threshold (Ct) values ranged from 28.6 to 40. Of 25 positive samples, 20 (80%) had a Ct value >35. The 4 most contaminated surface samples were the floor (7 of 55, 12.7%), the windowsill (4 of 20, 20.0%), the toilet seat (4 of 44, 9.1%), and the door handle (3 of 62, 4.8%). Other positive surfaces included bedside table (n = 2), bed rail (n = 1), respirator (n = 1), bench (n = 1), and fluids ramp (n = 1). Between the first and the third epidemic waves, the frequency of contaminated surfaces slightly decreased (10.0% and 5.5%, respectively; Fisher exact test: *P* = .13).

Contaminated rooms were compared to noncontaminated rooms for the variables shown in Table 2. The frequency of contamination was significantly higher in rooms of older patients.

**Table 2.** Variable Analysis According to Room Contamination

Variable	Contaminated Rooms	Noncontaminated Rooms	Total	*P* Value
Medical unit	12/14 (85.7)	32/47 (68.1)	44	.31
Intensive care unit	2/14 (14.3)	15/47 (31.9)	17	
Age, median y (IQR)	84 (72.2–92.2)	71 (57–83)	61	**.008**
Sex, male	9/14 (64.3)	33/47 (70.2)	61	.75
Symptoms present	11/14 (78.5)	40/47 (85.1)	51	.68
Symptom delay, median d (IQR)	7 (5–10)	10 (6–14)	51	.11
Admission delay median d (IQR)	4.5 (3.7–12.5)	3 (2–6)	61	.09
Diagnosis delay median d (IQR)	3.5 (2–5.5)	3 (2–9)	61	.82
Ct of clinical sample, median (IQR)	24.83 (20.13–27.42)	23.79 (20.06–28.93)	54	.75
Historical strain	3/9 (33)	13/37 (35.1)	16	.34
α (alpha) variant	3/9 (33)	19/37 (51.4)	22	
β (beta) variant	3/9 (33)	5/37 (13.5)	8	
Mechanical ventilation	8/14 (57.1)	36/47 (76.1)	44	.18
Intubation	1/8 (12.5)	4/36 (11.1)	5	.87
Nasal	3/8 (37.5)	15/36 (41.7)	18	
Mask	3/8 (37.5)	9/36 (25)	12	
Optiflow	1/8 (12.5)	8/36 (22.2)	9	
Flow ≥6 L oxygen	4/7 (57.1)	15/32 (46.9)	19	.69
Oxygen flow. L	7 (2–13)	4.5 (2.25–14.25)	39	.63
HA COVID-19	4/14 (28.6)	9/47 (19.1)	13	.47

Note. Ct, cycle threshold value; IQR, interquartile range; HA, hospital acquired. Quantitative values were calculated using the Mann-Whitney test as median (IQR) and qualitative values were calculated using the Fisher or χ^2^ test.

Significant *P* value is shown in bold.

## Discussion

The frequency of surface contamination (7.1%) in our study was lower than that reported in other studies (533 of 3,077, 17.3%), although surface contamination has varied from 0% to 74.2% across published studies.^3^ Furthermore, most positive samples have high Ct values, suggesting a low genome level and a likely lack of infectivity in cell culture, as shown by others.^3,7^

Interestingly, 4 rooms (nos. 1, 2, 3, and 52) were more contaminated than others (at least 3 positive environmental samples per room), suggesting either the presence of a “super-spreader” patient or just a poor room cleaning (Table 1).

Only a single air sample was positive. This air contamination was lower than frequencies reported in other studies (82 of 471, 17.4%).^2^ The variations (from 0% to 100%) of air contamination might be explained by differences of sampling across studies (volume of sampled air, sampling technique, distance from the patient, areas of sampling, etc).^2,8^

We investigated risk factors for the environmental spread of SARS-CoV-2 (Table 2). The only significant risk factor was patient age. This might be explained by the higher severity of patients who required heavier care load resulting in more contacts with the healthcare staff. We did not find any association between environmental contamination and type of ward (medical versus intensive care units) despite a higher frequency of aerosol-generating procedures in ICUs.

Since the first wave of COVID-19, many variants of SARS-CoV-2 have emerged. The third wave was characterized by the emergence of the α (alpha) variant (20I/501Y.V1; B.1.1.7).^9^ Nevertheless, the environmental contamination frequency between historical strain and variant was not significantly different. The high spread of the α variant does not seem to be explained by environmental contamination.

This study had several limitations. First, the correlation between the Ct values of clinical and environmental samples was difficult to interpret due to the different PCR assays used. Second, the viral culture for infectivity determination was not done, but previous studies failed to isolate the virus in cell culture in samples with Ct > 35.^10^ Only 2 of 16 studies have successfully isolated viable virus in cell culture from the environment.^2,3^ Third, we did not assess the quality of room cleaning, although we insured that sampling was performed at a distance from any cleaning procedure.

In conclusion, The frequency of surface and air contamination by SARS-CoV-2 was low and decreased by 50% when comparing the first epidemic wave to the third one, despite the emergence of more transmissible virus variants. The environmental spread of SARS-CoV-2 is likely limited in the hospital setting.
